# In vitro screening of EPS‐producing *Streptococcus thermophilus* strains for their probiotic potential from Dahi

**DOI:** 10.1002/fsn3.2843

**Published:** 2022-03-24

**Authors:** Robina Taj, Tariq Masud, Asma Sohail, Shehla Sammi, Rooma Naz, Bal Kumari Sharma Khanal, Malik Adil Nawaz

**Affiliations:** ^1^ Institute of Food and Nutritional Sciences PMAS Arid Agricultural University Rawalpindi Rawalpindi Pakistan; ^2^ 384987 Department of Food Science and Technology The University of Haripur Khyber Pakhtunkhwa Pakistan; ^3^ Abbasyn University Islamabad Campus Islamabad Pakistan; ^4^ Ministry of Agriculture and Livestock Development Government of Nepal Singhadurbar, Kathmandu Nepal; ^5^ 2221 Agriculture and Food Commonwealth Scientific and Industrial Research Organisation Werribee Victoria Australia

**Keywords:** antibiotic, cell surface hydrophobicity, cellular auto‐aggregation, Dahi, exopolysaccharide (EPS), gastrointestinal transit tolerance

## Abstract

Dahi is a very common and traditional fermented dairy product in Pakistan and its neighboring countries, it represents a rich source for the isolation of many new strains of lactic acid bacteria (LAB). The major objective of this study was to evaluate the probiotic potential of novel exopolysaccharide (EPS)‐producing strains of *S. thermophilus* isolated from Dahi, sold in the local markets of Rawalpindi and Islamabad, Pakistan. In this study, 32 isolates of *S. thermophilus* were initially isolated from Dahi and out of these, 10 identified strains were further screened for their EPS‐producing ability. Maximum EPS production was estimated for RIY strain (133.0 ± 0.06), followed by RIH4 strain (103.83 ± 0.76) and RIRT2 strain (95.77 ± 0.22), respectively. Thereafter, in vitro studies revealed that these newly identified EPS‐producing strains of *S. thermophilus* fulfilled the basic requirements for probiotic functions; including resistance to harsh conditions of GIT, good cell surface hydrophobicity, auto‐aggregation, and co‐aggregation, especially against *L. monocytogenes*. Finally, the safety assessment displayed that these strains were also sensitive to clinical antibiotics, including vancomycin. Thus, these selected EPS strains of *S. thermophilus* act as potential candidates for biostabilizers in the preparation of consumer‐friendly fermented probiotic milk products.

## Graphical abstract

Dahi is a famous fermented dairy product in Pakistan used for LAB isolation. *S. thermophilus* EPS‐generating strains were isolated from Dahi obtained in the marketplaces of Rawalpindi and Islamabad, Pakistan. Ten *S. thermophilus* strains were chosen for EPS production testing. This was followed by selecting three strains RIY (133.00.06), RIH4 (103.830.76), and RIRT2 (95.770.22). We chose these three strains based on their antibiotic susceptibility and acid/bile tolerance. In RIRT2, *L. monocytogenes* ATCC19115 survived at 0.3 and 1.5 percent bile. RIY exhibited the best acid tolerance (69%) and survival (25%), followed by RIH4 (nearly 65%) and RIH5 (almost 20%). RIY had the highest bile tolerance (0.3%), followed by RIH4 (80%) and RIRT2 (>70%). On the heels of RIRT2, RIY (57.7B) and RIH4 (97.80.4). (56.8C). Antibiotic resistance was found in all three.

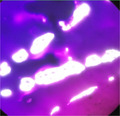

## 1 INTRODUCTION

*Streptococcus thermophilus* is a common LAB with intrinsic functional characteristics, such as technological, organoleptic, and nutritional properties, which are considered important in the food and dairy industry (Quigley et al., 2013). This microorganism is not only able to produce lactic acid by a fermentation process but also with EPS that can act as a bio‐stabilizer in yogurt, contributing to texture, firmness, and viscosity of the final product (De Vuyst et al., 2003). In order to reduce syneresis and enhance the consistency of yogurt, hydrocolloids or synthetic stabilizers have been used in the past. These stabilizers were mostly chemically modified and declared banned in some countries. However, consumer demand for cost‐effective, free from synthetic additives, natural, cholesterol‐lowering, and diabetic‐friendly products has increased. These problems can be overcome, and consumer demands can be fulfilled, by using EPSs as a viable alternative (Feldmane et al., 2013). Due to this capability EPSs can be used in fermented foods as a natural emulsifier or food‐grade hydrocolloid (Ruhmann et al., 2015). In addition to these functional properties, this bacterium is reported to have many health benefits for animals and humans, and numerous strains of *S. thermophilus* have been known to possess probiotic properties. Probiotics are briefly defined by WHO/FAO (2006) as “live micro‐organisms which when administered in sufficient amounts (i.e., minimum 10^6^ CFU gm/L) (Shah, 2007) give their consumer or host a specific health benefit.” They have either a direct or indirect impact on the gastrointestinal tract; including immune‐modulation, mitigation of diarrhea due to miscellaneous causes, and prophylaxis of gastrointestinal infections. Probiotics are also effective against various intestinal diseases such as *Helicobacter pylori* infections, colon cancer, and inflammatory bowel disease (Marteau et al., 2001; Tuncer & Tuncer, 2014), as well as for lactose intolerance, blood cholesterol, bacterial vaginosis, and atopic dermatitis (Shah, 2007).

Probiotic strains must possess some basic characteristics including survival in simulated gastrointestinal (GI) tract conditions, antibacterial activity, cell aggregation, and cell surface hydrophobicity or bacterial adhesion to hydrocarbons (BATH), as a measure of intestinal colonization ability against adhesion of enteropathogens (Monteagudo‐Mera et al., 2019). Previously, the higher colonization was observed for high hydrophobicity (De Souza et al., 2019; Miljkovic et al., 2015, 2019). Essentially, BATH is associated with the adherence of strains and aggregation is the clumping of cells associated with their persistence in the GI tract (Saito et al., 2019). These properties are also required for probiotic starter culture development (Guarner et al., 2005; Miljkovic et al., 2015, 2019; Vinderola & Reinheimer, 2003).

A commonly used domestic dairy product in Asian countries including Pakistan is “Dahi”; an indigenous yogurt containing a mixture of LAB strains with *Lactobacillus bulgaricus* and *Streptococcus thermophilus* as major microbiota. Previous studies have confirmed the presence of LAB, including *Lactobacillus bulgaricus* (Ali et al., 2019 *), Lactobacillus acidophilus* (Farid et al., 2021), and *S. thermophilus* (Mahmood et al., 2013) in Dahi samples collected from the markets of Rawalpindi and Islamabad, Pakistan.

These microorganisms have antibacterial activity against pathogenic bacteria and prevent gastrointestinal infections in the consumers, presenting probiotic effects (Mahmood et al., 2013; Soomro & Masud, 2012). However, studies related to the isolation of EPS‐producing strains of *S. thermophilus* from Dahi, with well‐studied characteristics and probiotic features, are limited. Therefore, the aim of this study was to assess the probiotic potential of new EPS‐producing strains of *S. thermophilus*, with biostabilizing effects, obtained from a Dahi source which can be used in consumer‐friendly dairy products.

## 2 MATERIALS AND METHODS

Dahi samples (*n* = 101) were collected from the local markets of Rawalpindi and Islamabad. These samples were collected under aseptic conditions and taken immediately to the laboratory for analysis.

### 2.1 Isolation and identification of *S. thermophilus*

The selective medium M17 (CM0817) Oxoid England was used to recover isolates of *S. thermophilus* from Dahi samples. M17 agar medium (composed of 3.725g M17 broth, 1.5% Technological agar and 10% lactose) was prepared in 100ml distilled water according to the instructions of the manufacturer; pH was adjusted to 6.9 with 6N NaOH, mixed on a magnetic stirrer and sterilized in a digital autoclave at 121°C for 15 min. (Hirayama, Japan). The agar was then poured into sterilized Petri dishes and allowed to solidify. Inoculation of collected samples was performed by the streaking method, on M17 agar plates, and incubated at 37°C for 24–48 h. The obtained isolates were tested through Gram staining and only Gram (+) colonies were further screened based on their morphological and biochemical properties, according to Buchanan and Gibbons (1974) and as mentioned in Bergey’s manual. The selected colonies were then further analyzed and identified using the API kit method. For this purpose, API (Analytical Profile Index) 50CHL (API System, BioMerieux, France) was used according to the instructions given by its producer. Finally, *S. thermophilus* was confirmed at the molecular level with polymerase chain reaction (PCR) by amplifying the 16S rRNA region of these isolates using specific primers 5ˋ ACGCTGAAGAGGAGCTTG 3ˋ and 3ˋ GCAATTGCCCCTTTCAAATA 5ˋ according to the standard method of Tab asco et al. (2007) with some modifications.

### 2.2 Exopolysaccharide production of strains

The identified and characterized *S. thermophilus* strains were further evaluated for having exopolysaccharide production ability. Initially, the production of EPSs on skim milk (Oxoid) plus nutrient agar (Oxoid) medium plates after incubation (37°C for 24 h) were visually observed by checking ropiness, mucidness, or capsulation of the strains using a sterile wire loop (or toothpick, like in Zivkovic et al., 2015) (Ali et al., 2019; Muigei et al., 2013; Paulo et al., 2012). Finally, mucoid or ropy (EPS producing) colonies of *S. thermophilus* were selected and further assessed for their EPS production.

In order to estimate EPS production, strains were inoculated (2%) in sterilized fermentation medium and incubated at 42°C for 24 h. 100ml of fermentation medium was prepared by adding 7ml of 11% skim milk (LP0031) Oxoid England, 3.0g nutrient agar (Oxoid, England), and 1.0g tryptone (Oxoid, England) in distilled water to make the volume up to 100ml, mixed on magnetic stirrer, and autoclaved at 121°C for 15 min.

#### 2.2.1 EPS isolation

Exopolysaccharides were isolated from the fermented medium according to the method described by Rimada and Abraham (2003), with slight modifications. The fermented sample (100ml) was taken and heated to boiling point (100°C) in a water bath for about 15 min. in order to remove proteins (to inactivate enzymes) and polysaccharides attached to the cell walls. After cooling to room temperature, the sample was centrifuged at 8,000 rpm for 10 min. to remove the cells. 17ml of 85% trichloro‐acetic acid (TCA) was then added to the sample (100 ml), cooled to 4°C, and centrifuged again at 8,000 rpm for 10 min. EPS concentration in the supernatant was increased by precipitation with cold ethanol (−20°C), with a 1:3 concentration and stored overnight at 4°C. The final precipitate, obtained by centrifugation at 8000 rpm for 10 min., was dissolved in distilled water (100ml) and stored at 4°C. The collected pellets of EPS were again suspended and filtered through a dialysis tube (molecular weight cut‐off 8–14 KDa, Beijing Solarbio Science & Technology Co., Ltd., China). The dialysis was performed against water for 48h., with water removal after every 8th hour. For further quantity determination, the solutions were prepared according to the method of Xu et al. (2010).

#### 2.2.2 EPS quantification

EPS quantification was carried out according to the method of Dubois (Dubois et al., 1956) based on the phenol‐sulfuric acid method, with slight modifications. Firstly, 5% of phenol red solution was prepared in the distilled water then 2ml of sample (EPS solution) and 1ml of phenol solution were mixed in the test tube. 5ml of concentrated sulfuric acid was added to the mixture and left for 10 min. Then, the mixture was shaken by vortex and incubated at 30ºC for 10 min. (until development of a yellow‐orange color). The control was prepared by adding 400µl phenol solution in 400µl of distilled water. Afterward, the absorbance of samples was measured by spectrophotometer (UV‐9200) at 490nm, and readings were compared with the control to measure total carbohydrate content. The amount of EPSs in each sample was interpreted by using glucose standard calibration line and expressed as mg/L glucose equivalent. Calibration line is prepared by glucose solution (1mg/ml) as standard, using 6 different proportions as defined by Feldmane et al. (2013) and Muigei et al. (2013).

#### 2.2.3 Evaluation of technological properties of EPS‐producing strains

The strains identified as EPS producers were used to ferment milk in order to determine technological properties, such as titratable acidity, curdling time, flavor, body, and texture of the curd. Sensory attributes (flavor, body, and texture) were determined through sensory evaluation method and titratable acidity by titration method. All the experiments were conducted in triplicates.

### 2.3 Antibacterial activity of *S. thermophilus* strains

For measuring antibacterial activity, pathogenic strains *E. coli* ATCC25922, *S. aureus* ATCC6538, *P. aeruginosa* ATCC25923, and *L. monocytogenes* ATCC 19,115 were obtained from the Department of Pathology, Pakistan Institute of Medical Science (PIMS) (Mahmood et al., 2013). The stocks of all the strains were maintained in 20% (v/v) glycerol and stored at −80°C.

For this purpose, the paper disc method was used, as described by Soomro and Masud (2012), with slight modifications. Sterilized paper discs of 6‐mm diameter made of Whatman filter paper no. 1, were kept on nutrient agar plates having a target pathogenic strain, whereas discs carried an adsorbed aliquot (20µl) of cell‐free supernatant. pH of the nutrient agar medium was adjusted to 7.2. To obtain a cell‐free supernatant, freshly overnight‐grown culture was attained in broth medium, and its pH was adjusted to 5.5 with 1 M NaOH. It was then centrifuged at 13,000 rpm for 10 min and the supernatant (cell‐free) was collected to send through a syringe filter (0.2µm) to remove bacterial cells. For comparison with the control, Ampicillin disc (10µg) was used as a reference antibiotic. The concentration of the overnight‐grown culture of indicator strains was adjusted according to 0.5 McFarland turbidity standard. The plates were then kept in an incubator for 24 h at 37°C. Resulting clear inhibition zones, formed around paper discs, were then measured for evaluation of antibacterial activity. Inhibition zones or spectra round discs were computed in diameter (mm).

### 2.4 Bile salt resistance and acid tolerance test

Bile salt tolerance and acid tolerance of isolates was conducted by the methods of Hassanzadazar et al. (2012) and Singhal et al. (2010), with slight modifications. For the acid tolerance test, M17 broth medium with adjusted pH values (2 and 3) was used to create in vitro acidic conditions of the gastrointestinal tract. pH 2 and 3 were adjusted with 1N HCl while pH 6.9 was adjusted to serve as a control. Overnight‐grown culture of *S. thermophilus* strains (1%) were then inoculated to M17 broth and incubated at 37°C for 5h. Percentage acid tolerance was found by measuring optical density (O.D.) at 600 nm using following formula:

$$\text{Survival}\;\left(\%\right)=\frac{O.D.(\text{pH}7)‐O.D.(\text{pH}2\;\text{or}\;3)}{O.D.(\text{pH}7)}\times 100$$

For bile salt tolerance, a fresh overnight‐grown culture of *S. thermophilus* strains (1%) was used for inoculation in M17 broth medium, supplemented with 0.3% and 1.5% bile salts (w/V), while M17 broth without bile salt (0%) supplementation was used as the control. Samples were incubated at 37°C for 6 h. and optical density (O.D.) was measured at 600 nm to determine the bile tolerance percentage of strains using the following formula:

$$\text{Survival}\;\left(\%\right)=\frac{O.D.(0\%\text{bile})‐O.D.(0.3\%\text{or}1.5\%\text{bile})}{O.D.(0\%\text{bile})}\times 100$$

### 2.5 Auto‐aggregation assay

Auto‐aggregation assay was performed in line with the method as outlined by Kaushik et al. (2009), with slight modifications. For this purpose, cell pellets from fresh growth of isolates were obtained by centrifugation (8000 rpm for 10 min.). The cell pellet was then washed and resuspended in 0.01 M phosphate buffer saline (PBS). Initial cell concentration (initial absorbance) was adjusted according to 0.5 McFarland standard at 600 nm and then incubated at 37°C for 2h. After 2 h, the suspension was centrifuged to obtain the cell pellet and mixed with the respective broth of equal volume removed. The supernatant was used to measure its absorbance (final absorbance) while the broth was used as the control. The following formula was used for calculating the percentage of auto‐aggregation capability.

$$\text{Auto}\text{‐}\text{aggregation}\left(\%\right)=\frac{\text{Abs}.(\text{initial})‐\text{Abs}.(\text{final})}{\text{Abs}.(\text{initial})}\times 100$$

### 2.6 Bacterial adherence to hydrocarbons (BATH) test

The method used, with some modifications, for determining percentage of bacterial adherence or hydrophobicity of *S. thermophilus* strains, was described by Kaushik et al. (2009). For this purpose, three different hydrocarbons (xylene, n‐hexadecane, and dichloromethane) were selected for measuring selected strains adherence percentage to these hydrocarbons. Briefly, fresh overnight‐grown culture was centrifuged (at 8000 rpm for 10 min.) to obtain the cell pellet. The cell pellet was then washed and resuspended in 2.5ml 0.01 M phosphate urea magnesium (PUM) buffer. Initial absorbance of cell suspension was set to 0.7 at 600 nm and then 1 ml of any tested hydrocarbon (xylene, n‐hexadecane, or dichloromethane) was added to the cell suspension. This suspension was then incubated at 37°C for 10 min. and vortexed (2 min.) to mix the two phases and again incubated at 37°C for 1 hr. After the incubation period, phases were separated and the aqueous phase was collected carefully to measure its absorbance at 600nm, using the following formula:

$$\text{Hydrophobicity(\textbackslash{}\% )}=\frac{\text{Abs}\text{.(initial)}‐\text{Abs}\text{.(final)}}{\text{Abs}.(\text{initial})}\times 100$$

### 2.7 Antibiotic susceptibility assay

The disc diffusion method (paper disc method) was used to determine the *S. thermophilus* strains’ susceptibility to antibiotics, as defined by Pisano et al. (2014), with some modifications. Antibiotics (10), which are currently used for the treatment of infections in the Allied hospitals of Pakistan, including Erythromycin (15 µg), Amoxicillin (10 µg), Vancomycin (30 µg), Kanamycin (30µg), Teicoplanin (30µg), Tetracycline (30 µg), Ciprofloxacin (5 µg), Streptomycin (10µg), Ampicillin (10 µg), and Gentamicin (10 µg), were selected for this test.

In this method, a bacterial lawn was prepared on agar plates with the concentration adjusted according to 0.5 McFarland standard and antibiotic discs were kept on it. These plates were then incubated at 37°C for 24 h and after 24 h clear zones or zones of inhibition (ZoI) were measured in diameters (mm) and compared with the interpretative zone diameters (CLSI M^100^–S21, 2011). The results were indicated as susceptible, moderately susceptible, or resistant.

### 2.8 Statistical study of data

The resulting data were statistically examined using Statistical package (SPSS 16.0 version). For this purpose, completely randomized design (CRD) was used and for graphical representation of the data Microsoft Excel was used. ANOVA (two way) followed by Tukey's test was also applied for statistical differences with a level of significance = 0.05 (Han et al., 2016).

## 3 RESULT AND DISCUSSION

### 3.1 Isolation and identification of *S. thermophilus*

One hundred and one samples of indigenous Dahi were collected from different areas of Islamabad and Rawalpindi to isolate *S. thermophilus* strains. On the basis of Gram staining, catalase test, and acid production, 76 isolates of lactic acid bacteria (LAB) were recovered and further characterized by morphological studies. Out of 76 strains, 44 were identified as bacilli Gram positive catalase negative, and 32 as cocci Gram positive and catalase negative. All selected isolates resulted negative for motility and spore formation ability as also reported by Sharma (2014).

In order to screen out *S. thermophilus* from 32 isolates of cocci, isolated Gram‐positive and catalase‐negative cocci were further differentiated on the basis of their growth at different temperatures and NaCl concentrations as well as their carbon dioxide gas production from glucose and confirmed through analytical profile index (API) test. Each experiment was conducted in triplicates and only promising isolates were further propagated for selection. The isolates which grew at 45°C but could not grow at even 2% NaCl concentration and did not produce carbon dioxide gas from glucose (homo‐fermentative), were selected. Sharma (2014) also used similar criteria of homofermentative bacteria for the screening of LAB isolates. Further biochemical testing through API confirmed that out of 32 LAB cocci, 20 isolates were *S. thermophilus*, three isolates were *S. cremoris*, and five were *L. lactis,* while only four isolates were identified as *Leuconostoc* spp. All 20 biochemically identified strains were subjected to PCR amplification of 16S rRNA regions. The specific primers used according to the sequence 5ˋ ACGCTGAAGAGGAGCTTG 3ˋ and 3ˋ GCAATTGCCCCTTTCAAATA 5ˋ published in NCBI gene bank and consequently isolates which gave PCR product of 200‐230bp were finally selected. These findings are similar to the results of PCR products of *S. thermophilus* previously described by Tab asco et al., (2007) and Mahmood et al., (2013). The PCR results confirmed the 10 selected strains as *S. thermophilus* (Ali et al., 2019; Kullen et al., 2000; Suhartatik et al., 2014).

### 3.2 Exopolysaccharide production of selected strains of *S. thermophilus*

#### 3.2.1 Screening of ropy and mucoid strains to assess EPS‐producing ability

**FIGURE 1:**
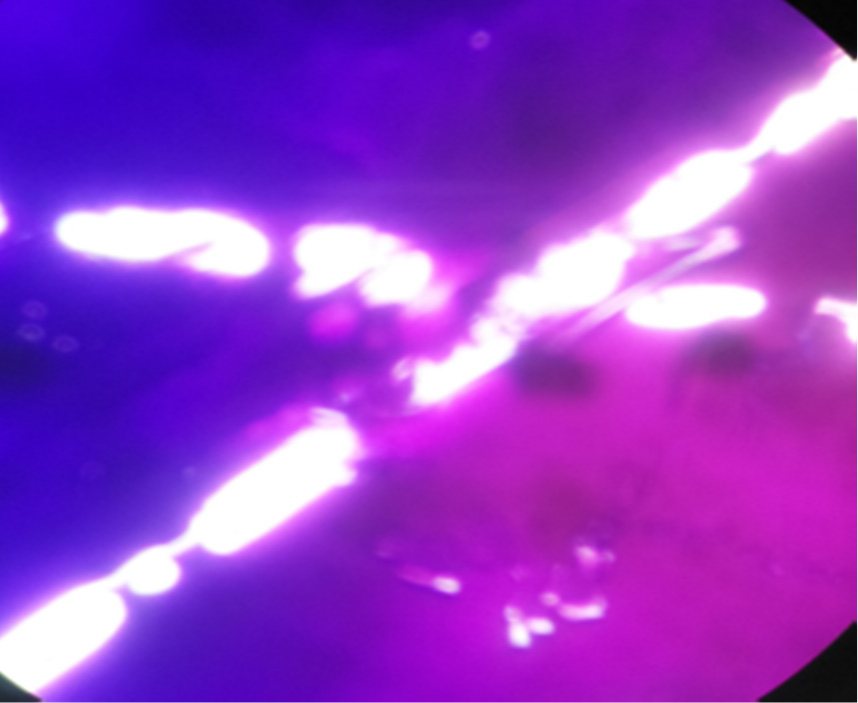
Capsule formation by *S. thermophilus* strains detected by staining method

**FIGURE 2:**
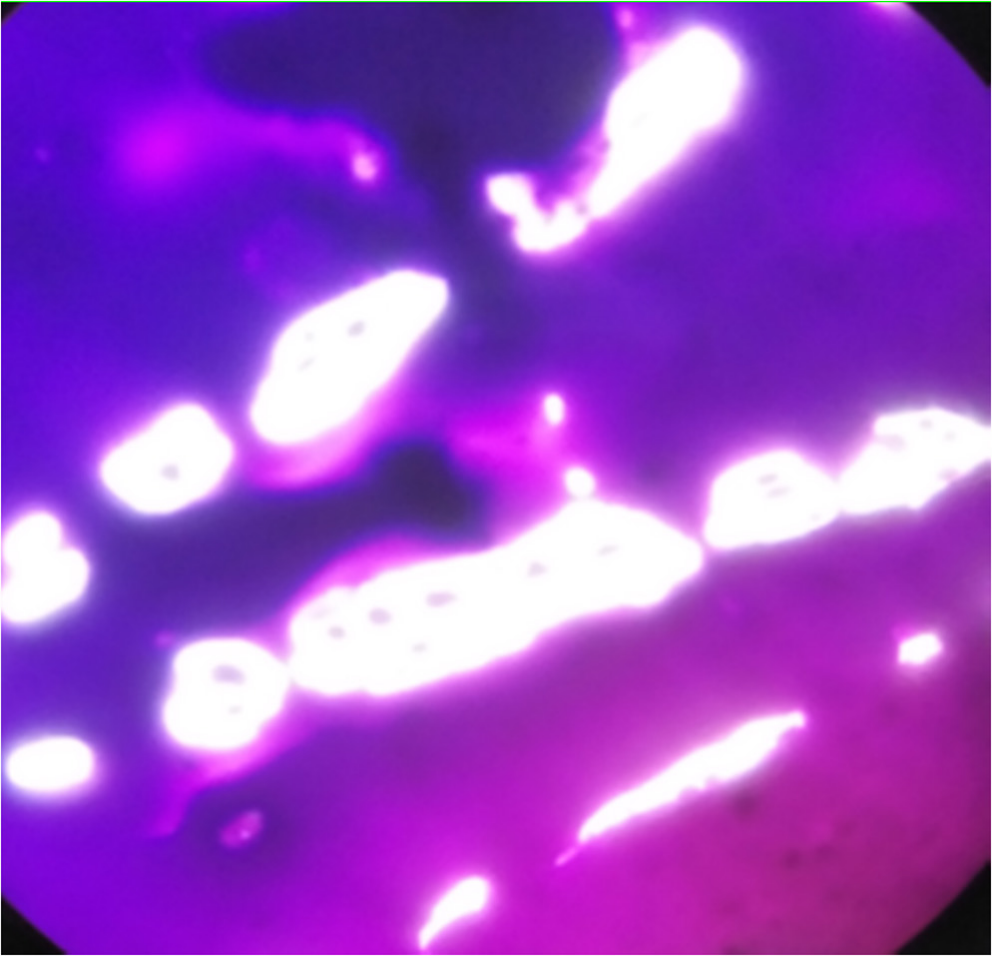
Detection of capsular extracellular polysaccharide produced by *S. thermophilus*

The 10 strains identified as *S. thermophilus* were tested for EPS production ability. For this purpose, initially their ropiness and mucoid nature was assessed through a visual observation method, that is, ropiness test. The strains which formed long ropy like structures when picked with a sterile inoculation wire loop, were considered as ropy strains. According to Gomez (2006) and Zivkovic et al. (2015), this phenotypic character can be associated with the production of exopolysaccharides on solid medium, however, exopolysaccharides can be capsular polysaccharides (CPS) or ropy polysaccharides (RPS). Capsulation was determined through staining with crystal violet and subsequently rinsing with 20% copper sulphate solution. The results obtained are shown in Table 1 and it can be seen that all ten selected strains showed positive mucoid colony growth on M17 and skim milk agar medium plates. However, two isolates (RIRT and RIR1L) did not show any ropy polymer production through visual observation while all others were positive for ropiness, including two isolates (RIY and RIH4) which were highly ropy. With regard to the capsulation‐forming ability, all the strains resulted positive to the capsulation test, except for the RIRT isolate. Capsule staining showed that some strains (RIH4) formed large and thick capsules while some formed relatively small or no capsules (Figure 1 and Figure 2). The disparity among different strains for production of ropy or capsular polysaccharides is due to different cultures and some strains even produced both ropy and capsular polysaccharides. Mozzi et al. (2009) also reported that ropy or capsular exopolysaccharide production is varied from culture to culture and hence some strains have the ability to form only one type of polymer, while others form both capsular and ropy polysaccharides.

**TABLE 1:** Assessment and quantification of EPS production by *S. thermophilus* strains isolated from local Dahi

Strain code	Type of EPS		EPS in skim milk media (mg/L) ± S.D.
	CPS	RPS	
RIY	+	++	133.0^a^ ± 0.06
RIK	+	+	93.17^d^ ± 0.28
RIM	+	+	27.43^h^ ± 0.40
RIRT2	+	+	95.77^c^ ± 0.22
RIH4	+	++	103.83^b^ ± 0.76
RIRT	‐	‐	19.67^j^ ± 0.57
RIHQ3	+	+	24.67^i^ ± 0.57
RIR1L	+	−	78.33^e^ ± 0.57
RIH1	+	+	37.67^g^ ± 1.52
RIH3	+	+	41.01^f^ ± 1.00

Note

All values are means of three replications and means carrying different letters are significantly different at alpha 0.05 (*p* <.05). Indications used are for highly ropy (++), less ropy (+), and nonropy (−) strains.

Mostefaoui et al. (2014) also used a similar mucoid and ropiness test for screening of EPS‐producing isolates after incubation for 48 h at 42°C. According to Ruas‐Madiedo and de Los Reyes‐Gavilan (2005), Welman et al. (2003), and Ricciardi et al. (1997) mucoidy of strains was assessed through appearance and visual observation of colonies’ growth and confirmed through an ethanol precipitation technique. According to Behare et al. (2010), the strains forming ropy polysaccharides were considered to be better than strains forming capsular EPS and, due to this, can be used in dairy industry as a biothickener.

#### 3.2.2 Exopolysaccharide isolation and quantification

EPSs produced by the tested strains (ropy or capsular) were further isolated and then quantified by the trichloroacetic acid method followed by precipitation through the cold ethanol method. A similar method was used by Han et al. (2016) for isolation and measuring the concentration of these polysaccharides.

The results obtained regarding EPS concentration are summarized in Table 1. It can be observed that different strains produced different amounts of extracellular polymers with a significant difference (*p* < .05) among all the tested strains. The selected strains were able to produce EPS in skim milk medium, from 19.67 to 133.0 mg/l. Stingele et al. (1996) that reported the presence in *S. thermophilus* SFI6 of *epsM* and *epsA* genes responsible of exopolysaccharides synthesis. Maximum EPS production was observed in RIY in skim milk medium (133.0^a^ ± 0.06) followed by RIH4 (103.83^b^ ± 0.76) while minimum EPS production was observed in RIRT (19.67^j^ ± 0.57). This variation in the results of EPS production might be attributed to the reason that exopolysaccharides production is dependent on the strains, which might be associated with the gene encoding on chromosome for EPS formation. In the literature it was reported that the total yield of EPS produced by the lactic acid bacteria (LAB) depends on the composition of the medium and conditions in which the organisms grow (i.e., medium, temperature, and incubation time) (Cerning et al., 1990). Gamar et al. (1997) also reported that EPS production and yield were influenced by the carbon source and concentration. Consequently, those strains which produced maximum quantity of EPS have a potential to replace the usage of chemical stabilizers in the dairy industry.

#### 3.2.3 Technological screening—a comparison of EPS‐producing strains

Technological properties including acidity, curdling time, body and texture of curd, and other sensory features are summarized in Table 2. As shown, EPS production greatly affects sensory evaluation, body, and texture of the curd. In detail, four strains (RIY, RIH4, RIK, and RIRT2) possess the highest ability in terms of short curdling time, high acidity production, and sensory attributes compared to the other isolates. Specifically, the strains RIRT, RIHQ3, and RIH1 produced acidic flavor while RIM, RIH3, and RIR1L exhibited moderate technological and sensory characteristics. Thus, it can be stated that EPS production affects technological parameters, specifically body and texture, by improving them. It can also be observed that there is an inverse relationship between acidity and curdling time but no relationship with the production of extracellular polymers was found. Thus, the four strains RIY, RIH4, RIK, and RIRT2, showing comparatively better results, can be used as potential candidates for yogurt making.

**TABLE 2:** Technological properties of EPS‐producing strains

Strain code	Acidity (%Lactic acid)	Curdling time (hrs)	Flavor, body, and texture
RIY	0.83 ± 0.01	5.0	Good body and texture, pleasant flavor
RIK	0.68 ± 0.02	7.0	Good body and texture, acidic flavor
RIM	0.63 ± 0.01	8.5	Good body and texture, mild flavor
RIRT2	0.71 ± 0.04	6.5	Poor body and texture, pleasant flavor
RIH4	0.78 ± 0.01	5.5	Good body and texture, pleasant flavor
RIRT	0.60 ± 0.03	9.0	Good body and texture, acidic flavor
RIHQ3	0.64 ± 0.02	8.5	Good mouth feel but acidic flavor
RIR1L	0.67 ± 0.02	7.0	Good body and texture, pleasant flavor
RIH1	0.66 ± 0.04	8.0	Good mouth feel but acidic flavor
RIH3	0.65 ± 0.01	7.5	Good texture, pleasant flavor

### 3.3 Probiotic potential of *S. thermophilus* strains

#### 3.3.1 Antibacterial activity of *S. thermophilus* strains

Our traditional fermented dairy product, Dahi, can be used as a source of probiotics because the microbial isolates included strains *of S. thermophilus*, which is identified as a probiotic bacteria (Bhowmik et al., 2009; Mahmood et al., 2013). In addition to the primary role of their milk acidification, these bacterial strains of *S. thermophilus* produce secondary metabolites such as antibacterial peptides and possess other probiotic features.

EPS‐producing strains were firstly investigated to ascertain their possible antimicrobial activity against food pathogens before determining other probiotic properties. Four pathogenic strains were used for this purpose (as shown in Table 3), namely *L. monocytogenes* ATCC 19,115, *E. coli* ATCC25922, *S. aureus* ATCC6538, and *P. aeruginosa* ATCC25923 as also previously used by Mahmood et al. (2013). Therefore, determination of the antibacterial activity of *S. thermophilus* strains against these indicator strains would be a novel character. It is revealed from the results that all the ten tested strains gave variable results and showed a wide range of antimicrobial activity against different pathogenic/indicator strains, having more or less zone of inhibition against one pathogen or more. These differences in the inhibitory activities of tested strains against different indicator strains may be due to their genotype or environmental factors.

**TABLE 3:** Antibacterial activity of cell‐free supernatants from *S. thermophilus* strains against different food pathogens

Cell‐free supernatant strain code	Indicator strain (Pathogen)			
	*E. coli* ATCC25922	*L. monocytogenes* ATCC19115	*P. aeruginosa* ATCC25923	*S. aureus* ATCC6538
RIY	−	++	++	+
RIK	++	++++	+++	++
RIHQ3	+	+++	−	++
RIM	+	+++	++	+++
RIRT	+	++	+	+
RIRT2	+	++++	+	+++
RIH1	+	+++	−	++
RIH3	−	+++	+	++
RIH4	−	+++	++	+
RIR1L	++	+++	+++	++

Note

Zone of inhibition (−) = no activity, (+) = visible inhibition, 1–4 mm (++), 5–12 mm (+++), ≥13mm (++++) (Mahmood et al., 2013).

The results of antibacterial activity of cell‐free supernatants, from *S. thermophilus* strains, are presented in Table 3. The maximum zone of inhibition (16mm) was observed against *L. monocytogenes* ATCC 19115 using RIK and RIRT2 supernatants, while *S. aureus* ATCC6538 was observed to be most sensitive on a maximum number of isolates, with a maximum zone of inhibition of 8 mm by RIM and RIRT2 supernatants. Mahmood et al. (2013) also reported maximum antibacterial activity against *L. monocytogenes* ATCC 19115 and *S. aureus* ATCC6538, by four *S. thermophilus* strains (S02FT, S03FT, S05FT, S06FT). Similar results were reported by Khalil (2009) who found that the crude extract of *S. thermophilus* CHCC3534 produced broad‐spectrum bacteriocin that is effective against *S. typhimurium* and *S. aureus*. Fontaine and Hols (2008) studied thermophilin 9, produced by *S. thermophilus* LMD‐9, that was active against other *S. thermophilus* and *L. monocytogenes* strains due to the presence of three mutative operons (bacSt operons) and the blpGSt gene, which encodes a putative modification protein to the inhibitory activity of LMD‐9.

*E. coli* ATCC25922 was found to be less sensitive to cell‐free supernatants from all the tested strains, with three strains (RIY, RIH4, and RIH3) showing no antibacterial activity or no detectable zone of inhibition. However, only a lower antibacterial activity was found by cell‐free supernatants of the remaining seven strains with two (RIK and RIR1L) having a zone of inhibition up to 4mm and five (RIRT, RIRT2, RIM, RIHQ3, and RIH1) having visible zones of inhibition (<1mm). Mahmood et al. (2013) also reported weak antibacterial activity (≤4mm ZoI) against *E. coli* ATCC25922 from cell‐free supernatants of 3 tested strains out of 11.

A mixed response was observed against *P. aeruginosa* ATCC 25,923 by cell‐free supernatants of the tested strains. The maximum zone of inhibition (12mm) was observed using RIK and RIR1L supernatant, while minimum zone of inhibition (<1mm) was observed using RIRT, RIRT2, and RIH3 supernatants. The supernatants of two strains (RIHQ3 and RIH1) showed no activity against *P. aeruginosa* ATCC 25923, while others showed medium (in between) zones of inhibition. Mahmood et al. (2013) stated that supernatants of only 3 strains out of 11 tested showed an inhibitory effect (≤8mm ZoI) against *P. aeruginosa* ATCC 25923.

#### 3.3.2 Acid and bile tolerance

##### Acid tolerance

If a minimum amount of 10^6^ log CFU (Nagpal et al., 2012) bacterial culture tolerates pH up to 2–3, it can be a potential candidate for a probiotic, as the initial pH of the stomach is 1.5 and it reaches up to pH 3–4 as food enters, which can remain for 4–5 h (Slavin, 2013).

**FIGURE 3:**
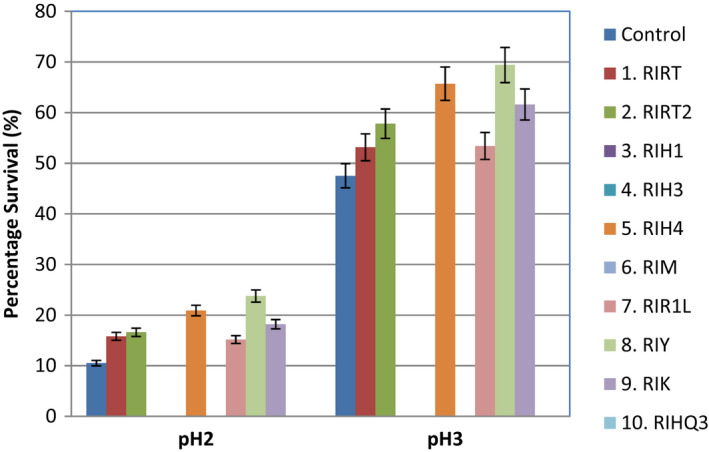
Survival to simulated acid conditions (pH 2 and pH 3) of *S. thermophilus* strains isolated from local Dahi (mean ± *SD*)

Low pH tolerance or acid tolerance of *S. thermophilus* strains was measured in vitro at two pH levels (pH 2 and pH 3). Only six strains out of ten selected were found to be tolerant to acid at both pH levels (2 and 3). The maximum tolerance under acidic conditions was observed in RIY with a 69% survival after 5h of incubation at pH 3 and 25% at pH 2, followed by RIH4, showing 65% survival at pH 3 for 5h and 20% at pH 2 (Figure 3). RIH4 is further followed by RIK with 62% survival at pH 3 and 19% survival at pH 2. Strain RIRT2 has 58% survival at pH 3 and 16% at pH 2 while strains RIRT and RIR1L showed almost similar survival rates at both pH levels with 52% survival at pH 3 and 15% at pH 2. Control strain gave survival percentages of 10% at pH2 and 47% at pH 3 as compared to other selected strains. Consequently, it can be said that pH 2 was more harmful for *S. thermophilus* than pH 3, however, the viability of cells declined during incubation. All six strains which remained viable at pH 3 had a survival rate of more than 50% and hence can be probable candidates as a probiotic culture (Liong & Shah, 2005).

Several studies have determined that *S. thermophilus* strains were unable to grow at low pH levels (Haller et al., 2001; Khalil, 2009; Mahmood et al., 2013; Maurad & Meriem, 2008); Tuncer and Tuncer (2014) reported that pH 1 was more lethal to *S. thermophilus* ST8.01 than pH 3, but during incubation at pH 3 viability of cells still declined and the percentage of inhibition was found to be more than 99.99%, whereas at pH 5 it was 95.43% and viability was retained. Another study by Mahmood et al. (2013) also reported that *S. thermophilus* strain S02FT was not capable to grow at pH 3.5 but maintained its viability at lower pH values. Our results are also correlated with the study of Khalil (2009) who found that *S. thermophilus* CHCC3534 was resistant to pH greater than 2 but nonresistant to 1.5. Some studies related to other probiotic bacteria also gave similar findings to our results. Maurad and Meriem (2008) reported that *L. plantarum* strains survived up to 6h after incubation at pH 2. According to Aswathy et al. (2008), LAB including *Streptococcus* growth increased at pH 5 and facilitated in the production of fermented vegetable and milk products.

##### Bile tolerance

Bile tolerance is one of the most essential criteria for a strain to be used as a probiotic culture (Hassanzadazar et al., 2012; Soleimanian‐Zad et al., 2009; Vizoso‐Pinto et al., 2006). Bile resistance and the ability of LAB to inhabit the intestinal tract appear to be correlated (Soomro & Masud, 2012). According to Aswathy et al. (2008), probiotic strains which are intended to be used for humans must have resistance to bile salts at 0.3% concentration.

**FIGURE 4:**
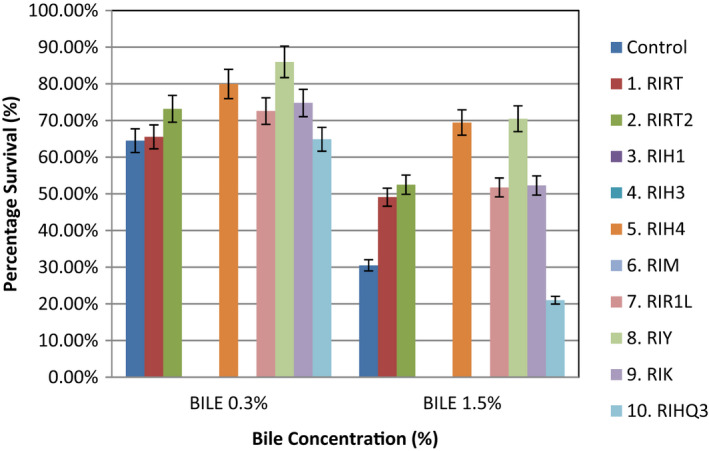
Survival to simulated bile conditions (0.3% and 1.5%) of *S. thermophilus* strains isolated from local Dahi (mean ± *SD*)

Bile tolerance of 10 selected EPS‐producing *S. thermophilus* strains was measured in vitro at two bile salt concentrations (0.3% and 1.5%). Seven strains out of ten were found to be tolerant at both concentrations. Maximum bile tolerance or survival percentage at 0.3% bile concentration was observed for RIY (about 85% survival), followed by RIH4 (80% survival) and RIRT2, RIR1L, RIK with >70% survival, while RIRT RIHQ3 had >60% survival. At 1.5% bile concentration maximum tolerance was observed for RIY (70% survival) followed by RIH4 (69% survival), RIRT2 (52% survival), RIR1L (51% survival), RIK (52% survival), and RIRT (49% survival) while least survival (21%) was observed for RIHQ3 (Figure 4). The control strain gave 64% survivability at 0.3% bile salt concentration and 30% at 1.5% as compared to other selected strains. Hence, all the seven strains (RIRT2, RIR1L, RIK, RIRT, RIHQ3, RIY, RIH4), which survived at 0.3% bile salt, fulfilled the criteria for being probiotic strains as reported by Boke et al. (2010). It is also described by Brashears et al. (1998) that 2% bile salt concentration is equal to that of the alimentary canal. Similar to our findings, Tuncer and Tuncer, (2014) found in their study that *S. thermophilus* strain ST8.01 was able to survive after incubation (24 h) at three different concentrations (0.3, 0.5, and 1%) of bile salt (w/v) and highest inhibition (38.34%) was observed at 1% bile salt concentration. Similarly, several other studies reported the bile tolerance of *S. thermophilus* strains even at 2% concentrations (Arias & Murray, 2009; Iyer et al., 2010). Mahmood et al. (2013) studied growth of *S. thermophilus* strain S02FT at three different concentrations (1, 2, and 3%) of bile salt and found that S02FT was able to survive at 2% concentration but unable to grow at 3%. However, Vinderola and Reinheimer (2003) reported much lower bile tolerance of *S. thermophilus* and stated that mostly strains were unable to survive at 0.5% bile concentration. From the mentioned literature, it can be stated that there is different growth at different bile salt concentrations, and this is probably due to a strain's specificity.

#### 3.3.3 Cell aggregation

**FIGURE 5:**
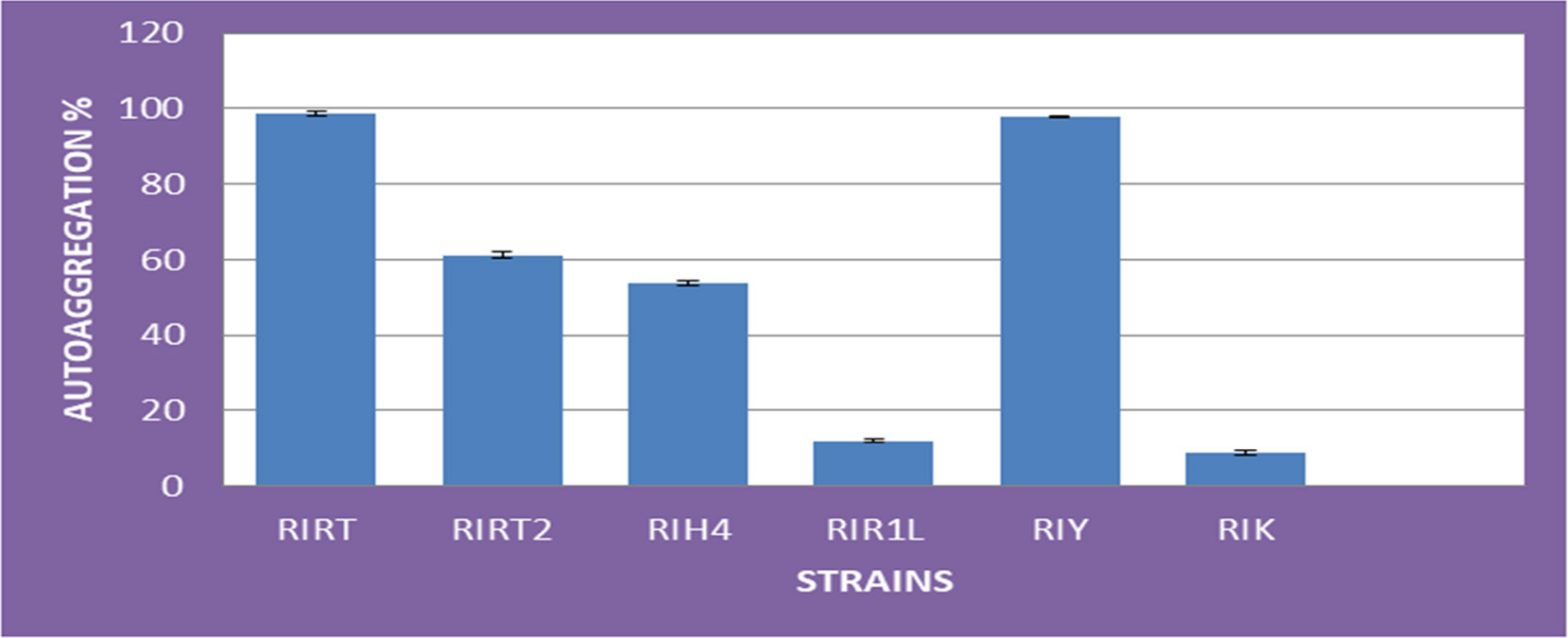
Auto‐aggregation (%) of *S. thermophilus* strains ± *SD*

Auto‐aggregation ability of probiotics is a prerequisite for their adherence with the epithelium cells of the intestine (Aslim et al., 2007; Collado et al., 2008). It can be seen from Figure 5 that the cellular aggregation percentage was variable for all the six selected strains. Maximum auto‐aggregation was found for RIRT (98.8 ± 0.6) followed by RIY (97.8 ± 0.4), RIRT2 (61.2 ± 1.0), and RIH4 (53.6 ± 0.6), respectively, while the minimum was observed for RIR1L (12.0 ± 0.5) and RIK (8.8 ± 0.6). These variations can be probably due to the auto‐aggregation ability of the single strain as also observed by other researchers (Kos et al., 2003; Todorov et al., 2009; Tuncer & Tuncer, 2014; Vlkova et al., 2008) reporting that physico‐chemical properties of cell surfaces such as hydrophobicity might have affected the auto‐aggregation abilities. The results in Table 4 show that high EPS‐producing strains exhibited more aggregation. Aslim et al. (2007) and Darilmaz and Beyatli (2012) also reported that high EPS‐producing strains exhibited significant aggregation. However, RIRT strain is less EPS producing but showed high auto‐aggregation ability which might be due to the strain specificity.

**TABLE 4:** Antibiotic sensitivity of *S. thermophilus* strains to different antibiotics

Strain code	AMP 10μg	AML 10μg	VA 30μg	TEC 30μg	TE 30μg	S 10μg	K 30μg	CN 10μg	E 15μg	CIP 5μg
RIRT	S(35)	I(15)	S(30)	S(25)	S(20)	R(0)	R(8)	I(14)	S(30)	R(0)
RIRT2	I(15)	S(30)	S(25)	S(25)	S(40)	I(12)	I(15)	I(15)	S(20)	I(14)
RIH4	S(20)	I(18)	S(25)	S(20)	S(30)	I(15)	I(13)	I(15)	S(40)	I(18)
RIR1L	I(12)	S(40)	S(35)	S(20)	S(25)	R(10)	R(0)	R(0)	S(20)	R(0)
RIY	S(20)	I(14)	S(26)	S(30)	S(40)	S(25)	S(20)	S(22)	S(30)	I(16)
RIK	S(30)	I(14)	S(40)	I(14)	S(20)	R(0)	R(0)	I(12)	S(40)	R(7)

Note

Zone of inhibition range 20mm or less, susceptible (S); 10‐19mm, Intermediate (I); 0‐10mm, resistant (R).

Abbreviations: AML, Amoxicillin; AMP, Ampicillin; Antibiotics E, Erythromycin; CIP, Ciprofloxacin; CN, Gentamicin; K, Kanamycin; S, Streptomycin; TE, Tetracycline; TEC, Teicoplanin; VA, Vancomycin.

Hence, the strains with aggregation percentages of 97.8 ± 0.4, 61.2 ± 1.0, and 53.6 ± 0.6 (Figure 5) are very interesting because their aggregating ability is higher than the reported aggregation of other *S*. *thermophilus* strains, except RIRT which is a high aggregating but low EPS‐producing strain. Tuncer and Tuncer (2014) reported 49.55 ± 6.24% auto‐aggregation of *S*. *thermophilus* ST8.01 strain. Some previous studies conducted also reported somewhat comparable auto‐aggregation percentages of LAB (Canzi et al., 2005; Koll et al., 2010; Rahman et al., 2008). Miljkovic et al. (2015) determined the important role of AggLb cell surface protein of strain *L. paracasei* subsp. *paracasei* BGNJ1‐64 for cell aggregation and collagen binding.

#### 3.3.4 Bacterial adherence to hydrocarbons (BATH)

The ability of bacteria to adhere to different hydrocarbons is the measure of the bacterial hydrophobicity to assess adherence of bacterial strains to the intestinal lining. Previously, in vitro analysis of bacterial adhesion to hydrocarbons using n‐hexadecane and xylene was carried out by Schillinger et al. (2005) and Kaushik et al. (2009), while using dichloromethane as a source of hydrocarbons was conducted by Jose et al. (2015).

**FIGURE 6:**
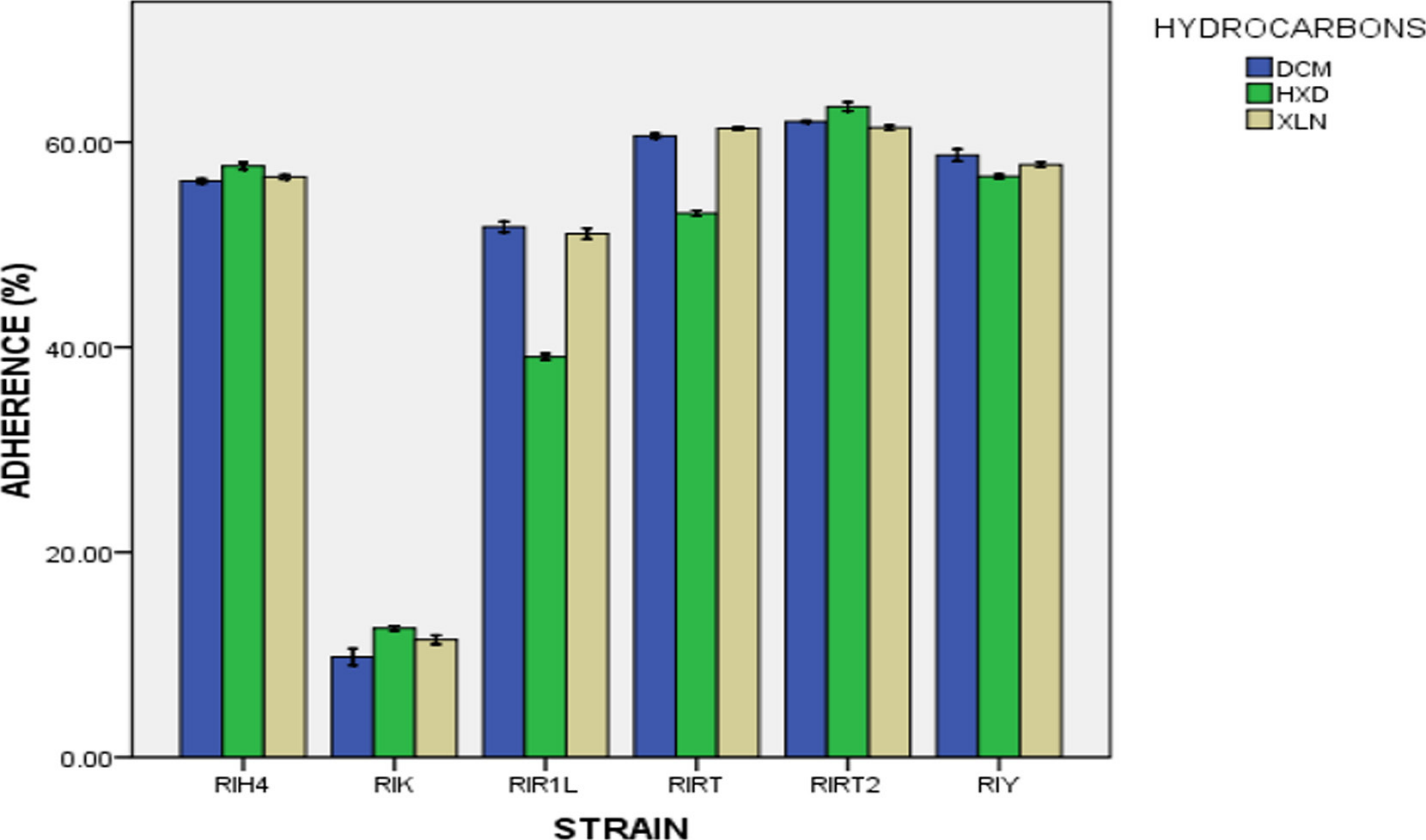
Adherence to different hydrocarbons of *S. thermophilus* strains isolated from local Dahi (mean ± *SD*)

In the present study, three hydrocarbons were used, namely n‐hexadecane, xylene, and dichloromethane (DCM) for testing the adherence percentage of selected strains of *S. thermophilus,* as shown in Figure 6. Among the three different hydrocarbons used, there is significant difference (*p* <.05) between n‐hexadecane and the other two hydrocarbons (xylene and dichloromethane), whereas between xylene and dichloromethane there is a nonsignificant difference (*p* >.05). In contrast to this study, Kaushik et al. (2009) reported that no significant difference was found in the adherence property of bacterial strains by using different hydrocarbons, while n‐hexadecane showed a lower mean value (47.1^B^) compared to xylene (49.9^A^) and DCM (49.8^A^). This decrease might be due to its toxic or destructive action on microbial cells. It can also be observed that among the adherence percentages of all the strains except RIRT (58.3^B^) and RIY (57.7^B^) there is a significant difference (*p* < .05). The maximum mean value was observed as 62.2^A^ for RIRT2 followed by RIRT (58.3^B^), RIY (57.7^B^), and RIH4 (56.8^C^), respectively, whereas the minimum adherence percentage was observed as 11.2^E^ for RIK. These differences might be due to the reason that cell surface hydrophobicity or adhesion of bacteria is basically a strain‐specific property which depends on the origin of the strain, its surface‐adhering or mucus‐adhering property (Grajek et al., 2016), or variation in cell surface protein expression levels along with the influence of the environment on the expression of certain proteins (De Vries et al., 2006). Previously, Tuncer and Tuncer, (2014) reported *S. thermophilus* ST8.01 adherence percentage to xylene as 67.23 ± 7.16% and similar results of higher adhering capacity percentage were observed in a study by Iyer et al. (2010). Although small differences exist for adherence percentage, the present study values are still higher than many other findings (Figure 6). According to the criterion as described by Tyfa et al. (2015) all the selected strains fall under the category of strongly hydrophobic except RIK, while RIR1L showed moderate hydrophobic behavior for hydrocarbon n‐hexadecane and strong hydrophobic behavior for xylene and DCM. It might be due to expression of surface proteins of cells or their preference for hydrocarbons (Draksler et al., 2004).

#### 3.3.5 Antibiotic susceptibility

A key requirement for probiotic strains is that they should not carry transmissible antibiotic resistance genes. Ingestion of bacteria carrying such genes is undesirable, as horizontal gene transfer to recipient bacteria in the gut could lead to the development of new antibiotic‐resistant pathogens (Guglielmetti et al., 2009; Saarela et al., 2000; Salminen et al., 1998). For this, the assessment of *S. thermophilus* strains’ susceptibility to clinically important antibiotics becomes important (Tuncer & Tuncer, 2014). The six selected strains were tested against 10 antibiotics by agar diffusion method as presented in Table 4. These strains were grouped as susceptible (S:20mm or >), resistant (R:0‐10mm), or intermediate (I:10‐19mm) to a particular antibiotic. It can be seen that the strains exhibited different behaviors to variable antibiotics as well as their concentrations. All the strains were found susceptible to Vancomycin (25‐40mm ZoI), Tetracycline (20‐40mm ZoI), and Erythromycin (20‐40mm ZoI) as well as to Teicoplanin (20‐30mm ZoI) with the exception of RIK strain which showed an intermediate effect (14mm ZoI). The strains RIRT, RIRT2, RIH4, and RIK exhibited intermediate response (12‐15mm ZoI), while RIY was susceptible (22mm ZoI) and RIR1L was resistant (0mm ZoI) against Gentamicin. Four strains RIRT, RIH4, RIY, and RIK showed intermediate responses (14‐18mm ZoI) to Amoxicillin except RIRT2 and RIR1L which showed susceptibility (30‐40mm ZoI). However, all strains were found susceptible (20‐35mm ZoI) to Ampicillin except RIRT2 and RIR1L which showed intermediate responses (12‐15mm ZoI).

The strains RIRT, RIR1L, and RIK were found to be resistant (0‐10mm ZoI) to Ciprofloxacin, Kanamycin, and Streptomycin while other strains showed intermediate (12‐18mm ZoI) to low susceptibility (20‐25mm ZoI). Similar results were reported by Katla et al. (2001), Temmerman et al. (2003), Aslim and Beyatli, (2004), Tosi et al. (2007), and Mahmood et al. (2013). These differences in the degree of inhibition with various antibiotics were possibly due to the difference in environment of strain isolation, as this is not the intrinsic feature of strains, or might be due to their different actions on the cell components such as the cell wall, protein and DNA synthesis, DNA gyrase, and RNA polymerase (Neu, 1992). Briefly, RIY showed susceptibility to the maximum number (against eight) of antibiotics, while RIR1L showed resistance to the minimum number (against three) of antibiotics. All other strains showed mixed response to different antibiotics. Mahmood et al. (2013) and Tosi et al. (2007) also found behavioral variations in the susceptibility patterns of *S*. *thermophilus* strains for different antibiotics. These behavioral changes in strains toward different antibiotics are due to the continuous exposure of strain culture to antibiotic‐resistant environments (Arias & Murray, 2009; Mathur & Singh, 2005).

Many previous studies also testified the susceptibility of *S. thermophilus* to several antibiotics, including E, chloramphenicol, CIP, TE, cephalothin, quinupristin, and reported medium susceptibility to highly resistant to CN, K, and S (Aslim & Beyatli, 2004; Katla et al., 2001; Temmerman et al., 2003; Tosi et al., 2007).

## 4 CONCLUSION

Today, the selection of probiotic, natural, EPS‐producing strains is gaining importance throughout the world for replacing artificial stabilizers. The present in vitro findings reflected that these three novel EPS‐producing strains of *S. thermophilus* (RIRT2, RIH4, and RIY), isolated from indigenous Dahi samples fulfill the basic criteria for the selection of probiotics with additional health benefits. Thus, these strains have a potential to be used as a source of biostabilizer starter culture for the different probiotic fermented milk products.

## CONFLICT OF INTEREST

The authors declare that they do not have any conflict of interest.

## Data Availability

The data that support the findings of this study are openly available in [repository name e.g “figshare”] at http://doi.org/ [doi], reference number [reference number].
